# Tissue-engineered 3D melanoma model with blood and lymphatic capillaries for drug development

**DOI:** 10.1038/s41598-018-31502-6

**Published:** 2018-09-04

**Authors:** Jennifer Bourland, Julie Fradette, François A. Auger

**Affiliations:** 1 Centre de recherche en organogénèse expérimentale de l’Université Laval/LOEX, Québec, Qc Canada; 2 Division of Regenerative Medicine, CHU de Québec–Université Laval Research Center, Québec, Qc Canada; 3 Department of Surgery, Faculty of Medicine, Université Laval, Québec, Qc Canada

## Abstract

While being the rarest skin cancer, melanoma is also the deadliest. To further drug discovery and improve clinical translation, new human cell-based *in vitro* models are needed. Our work strives to mimic the melanoma microenvironment *in vitro* as an alternative to animal testing. We used the self-assembly method to produce a 3D human melanoma model exempt of exogenous biomaterial. This model is based on primary human skin cells and melanoma cell lines while including a key feature for tumor progression: blood and lymphatic capillaries. Major components of the tumor microenvironment such as capillaries, human extracellular matrix, a stratified epidermis (involucrin, filaggrin) and basement membrane (laminin 332) are recapitulated *in vitro*. We demonstrate the persistence of CD31^+^ blood and podoplanin^+^/LYVE-1^+^ lymphatic capillaries in the engineered tissue. Chronic treatment with vemurafenib was applied to the model and elicited a dose-dependent response on proliferation and apoptosis, making it a promising tool to test new compounds in a human-like environment.

## Introduction

The incidence of melanoma is constantly rising in developed countries^1^ due to factors such as sunlight exposure and the use of tanning beds^2^. While being the less common skin cancer, melanoma is the deadliest due to its definite proclivity to metastasize. Therapies for metastatic melanoma are often not specifically adapted to these patients, thus new drugs are constantly developed to improve survival. One of the central issues with cancer therapy development is the dismal low rate of reproducibility between results observed in animal models and in the patients: less than 10% of data obtained from animal models can be translated to humans^3^. To reduce this attrition rate, improve preclinical screening and reduce the use of animals, more physiological human models are needed. Both monolayer and three-dimensional (3D) cell culture methods such as cancer spheroids are already used as tools for high-throughput screening^4,5^. The development of low to medium throughput models like tissue-engineered skin allows the production of 3D tissues partially reproducing either the native or the tumor microenvironment (TME)^6^. Most of the models available are based on synthetic or animal-derived scaffolds such as collagen of rodent or bovine sources in order to mimic the dermal extracellular matrix (ECM)^7–11^ or use decellularized human dermis, which have limited availability^12,13^. However, it is possible to avoid the use of animal-derived scaffolding elements by exploiting the ability of human stromal cells to produce their own ECM, thus recreating a more representative cutaneous microenvironment with relevant cell-matrix interactions^14–16^. Most *in vitro* tumor models imitate to a limited extent the crosstalk between the tumor and its microenvironment but none of them present a tissue-specific environment also including both blood and lymphatic capillaries.

Capillary networks are involved in tumor progression and are particularly important for melanoma modeling. Human microvascular endothelial cells (HMVEC) or human umbilical vein endothelial cells (HUVEC) can be incorporated in skin constructs, thus contributing to a more complex tumor microenvironment^17,18^. Their ability to form a network of blood capillaries which are stable, functional and perfusable has been observed in different types of skin substitutes produced using cell sheets, collagen-chitosan sponges, decellularized dermis or hydrogels^8,10,14,19–24^. Furthermore, lymphatic endothelial cells (LEC) can be isolated from HMVEC and incorporated into 3D constructs^25–29^. These LEC are able to assemble into lymphatic capillaries distinct from those formed by blood endothelial cells (BEC) in a collagen gel^8^. In a reconstructed dermis made of human ECM, LEC were shown to form a capillary network which can be modulated by growth factors such as hepatocyte growth factor and VEGF-C^25,30^.

The ability to produce blood and lymphatic vessels *in vitro* greatly facilitates the study of their interaction with tumor cells^31^. Other components contributing to the TME, such as fibroblasts and epithelial cells, should also be considered, as they influence tumor progression and invasion^32,33^. Here, we aimed to reproduce the melanoma microenvironment *in vitro* by engineering a more complete skin model presenting both blood and lymphatic capillaries. Key features of such a model should be to reproduce native tissue characteristics (structure and cell types) and to be responsive to known anti-melanoma therapeutic agents. The skin substitute was produced by the self-assembly method, an approach of tissue engineering based on the formation of ECM-rich cell sheets through the stimulation of fibroblasts with ascorbic acid^34^. It allows the production of human skin substitutes (epidermis and dermis) whose clinical relevance has already been well demonstrated by our group^35,36^. We incorporated human microvascular endothelial cells to recapitulate the formation of blood and lymphatic capillaries in the dermal compartment, and then melanoma spheroids were added to the tissue. These tumor models were then characterized and chronically treated with vemurafenib. Vemurafenib (Zelboraf ®) is a targeted therapy frequently used in melanoma with the BRAF-V600E mutation. It is a selective inhibitor of BRAF and thus induces tumor cell apoptosis^37,38^. We found that our model responded adequately to treatment regarding the proliferation and apoptosis of tumor cells, but also by recapitulating changes of tumor morphology.

## Results

### Formation of two distinct blood and lymphatic networks *in vitro*

**Figure 1:**
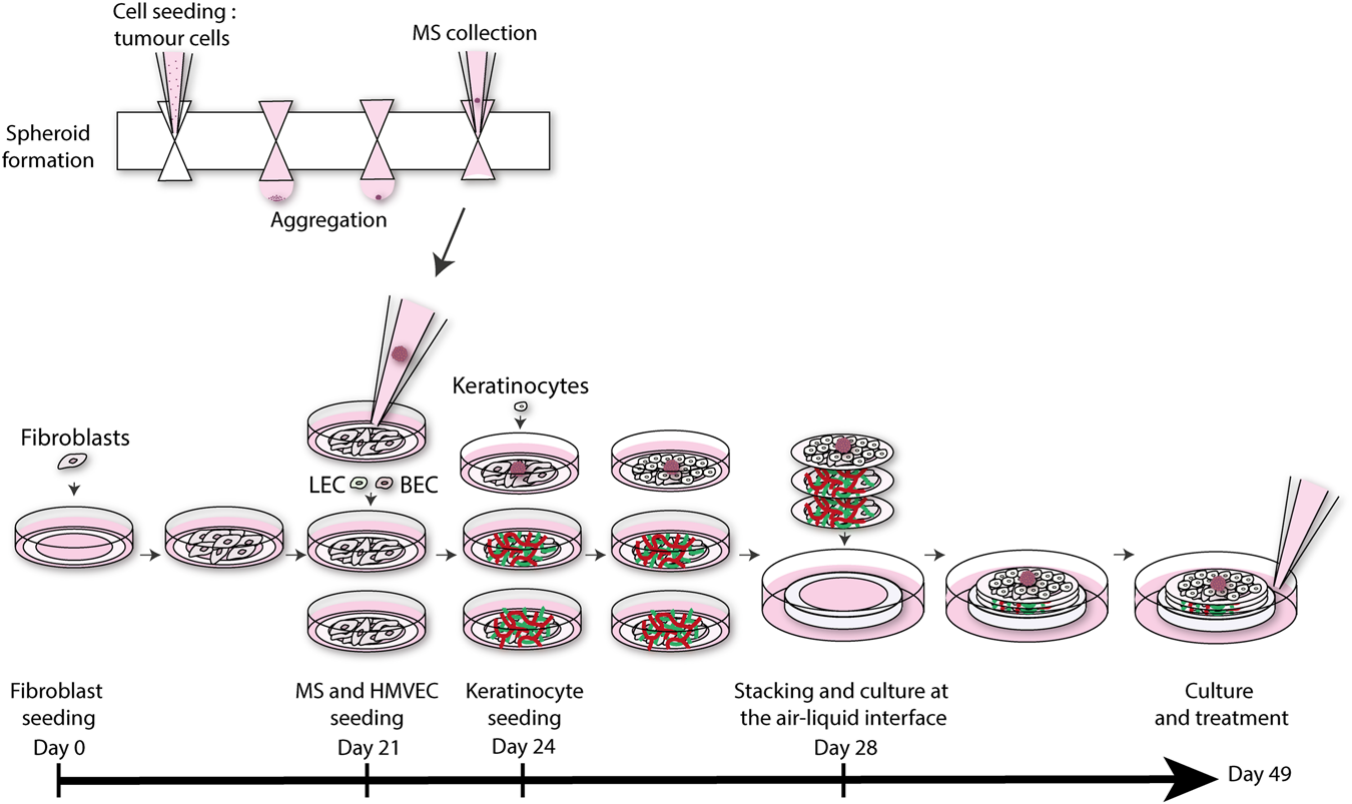
Method of production of the 3D microvascularized skin melanoma model. Fibroblasts were first seeded in tissue culture plates with peripheral paper anchors in presence of ascorbic acid to stimulate cell sheets formation and facilitate their manipulation. Endothelial cells were then added on two fibroblast cell sheets, while spheroids and keratinocytes were added on a third cell sheet. Three cell sheets were stacked after 28 days of culture and placed at the air-liquid interface. The complete models were considered mature (stratified epidermis, incorporated tumor and well-formed capillary networks) at day 38 and maintained in culture until day 49 (equivalent to 21 days at the air-liquid interface). Melanoma spheroids were formed in parallel using a GravityPLUS^TM^ plate. HMVEC: human microvascular endothelial cells; MS: melanoma spheroid; LEC: lymphatic endothelial cells; BEC: blood endothelial cells.

The ability of HMVEC to form capillaries was assessed in reconstructed skin produced by the self-assembly approach (Fig. 1).

**Figure 2:**
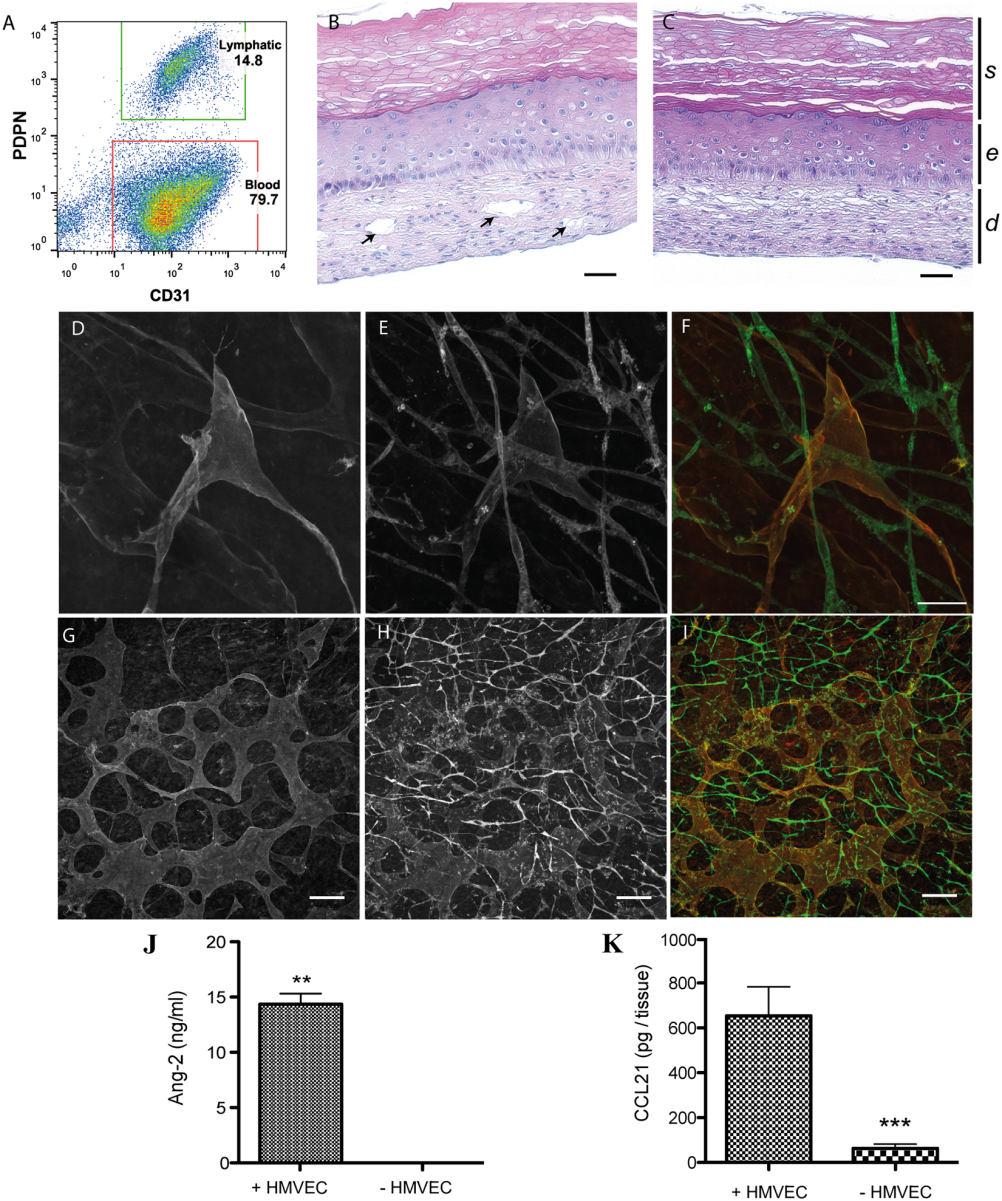
HMVEC assemble into blood and lymphatic capillaries in the 3D melanoma model. (**A**) Representative flow cytometry analysis of the cultured foreskin HMVEC population indicating the presence of two distinct populations (BEC and LEC) before their incorporation into the reconstructed skin. (**B**) Lumens (arrows) were observed after hematoxylin and eosin staining of HMVEC-enriched skin sections, but not in (**C**) non-endothelialized skin (scale bars: 50 µm). *s: stratum corneum*, *e*: epidermis, *d*: dermis. (**D**–**I**) Immunolabelings revealed the presence of two networks after staining for (**D**,**G**) podoplanin (PDPN, red signal) and (**E**,**H**) CD31 (green signal), **F** and **I** represent merge signals (scale bars: **D**–**F**: 50 µm, **G**–**I**: 150 µm). HMVEC specifically secrete (**J**) angiopoietin-2 (ANG-2) and (**K**) CCL21 in the reconstructed skin. **p-value ≤ 0.01, ***p-value ≤ 0.001. Statistical analysis: Student t-tests.

To evaluate the ability of foreskin HMVEC to generate both blood and lymphatic capillaries, the presence of BEC and LEC subpopulations was assessed by flow cytometry. At passage 4, cell suspensions from different donors (n = 4) all presented two distinct endothelial cell subpopulations: a population considered to be LEC, which is positive for podoplanin (PDPN) and CD31, and a population of BEC with a high expression of CD31 while negative for PDPN. Within the endothelial cell population, 14.8% were positive for podoplanin and considered lymphatic endothelial cells and 79.7% were considered blood endothelial cells (Fig. 2A). These subpopulations were also observed in cultures since distinct BEC and LEC colonies were formed (Suppl. Fig. 1). Once seeded onto cell sheets composed of dermal fibroblasts which are later combined to form the reconstructed skin with or without melanoma cells (Fig. 1), these HMVEC reorganized to form capillary-like structures as shown by the presence of lumens of different sizes (Fig. 2B) on sections stained with hematoxylin and eosin compared to non-endothelialized tissues (Fig. 2C). Immunolabelings revealed that two distinct networks were present: one strongly positive for CD31 and negative for PDPN (Fig. 2D–I), and a second one positive for PDPN with a weaker CD31 staining (Fig. 2D–I). The latter was also labeled by an anti-LYVE-1 (lymphatic vessel endothelial hyaluronan receptor 1) antibody (Suppl. Fig. 2). Lymphatic vessels presented an average diameter 3.9 times larger than for blood vessels (lymphatic vessels: 26.1 µm ± 3.7, blood vessels: 6.6 µm ± 0.8, p < 0.001), as measured on immunostained cryosections. In the microvascularized skin model, the secretion of chemokine (C-C motif) ligand 21 (CCL21) and angiopoietin-2 (ANG-2) was significantly increased in presence of HMVEC (Fig. 2J,K), indicating that the capillary networks were metabolically active.

The skin produced by the self-assembly method presented a dermis enriched in human ECM components, a differentiated epidermis featuring a proliferative basal layer and multiple suprabasal layers resulting in a *stratum corneum*, mimicking the human skin morphology (Fig. 2B,C). Melanoma spheroids were incorporated into this particularly biomimetic environment to generate a 3D melanoma model (Fig. 1).

### Melanoma tumor spheroids integrate in the cutaneous microenvironment

**Figure 3:**
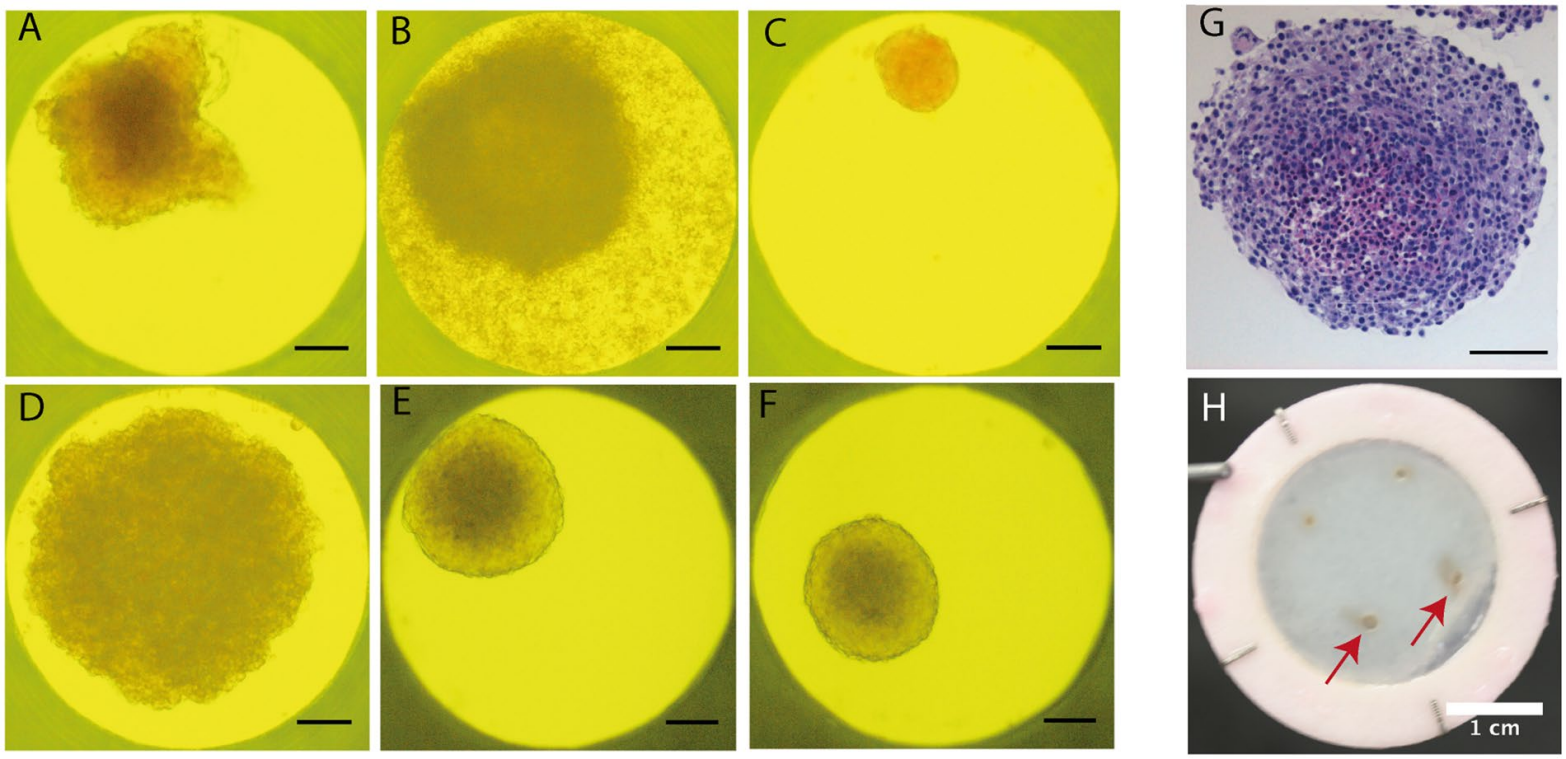
Formation of melanoma spheroids using the hanging drop method. Spheroids formed by (**A**) Malme 3 M, (**B**) A375, (**C**) RPMI 7951, (**D**) SK-MEL 28, (**E**) WM983A, (**F**) WM983B cell lines after 14 days of culture in GravityTRAP^TM^ plates. (Scale bars: 100 µm). (**G**) Hematoxylin and eosin staining of paraffin embedded spheroid section showing heterogenous composition and formation of compact spheroids by WM983A cells (Scale bar: 100 µm). (**H**) Tumor spheroids incorporated to skin tissues can easily be located (red arrows, Malme 3 M cells). Scale bar: 1 cm.

To generate a representative melanoma model, spheroids were generated using the hanging drop method and added to the reconstructed skin substitutes. Spheroids were obtained with A375, Malme 3 M, SK-MEL 28, RPMI 7951, WM983A and WM983B cells, each revealing a specific behavior prior to their incorporation into the skin substitutes to generate a melanoma model (Fig. 3, and Suppl. Table 1). They were integrated into the microvascularized skin substitutes by adding them on a cell sheet prior to keratinocyte seeding and cell sheet stacking (Fig. 1).

**Figure 4:**
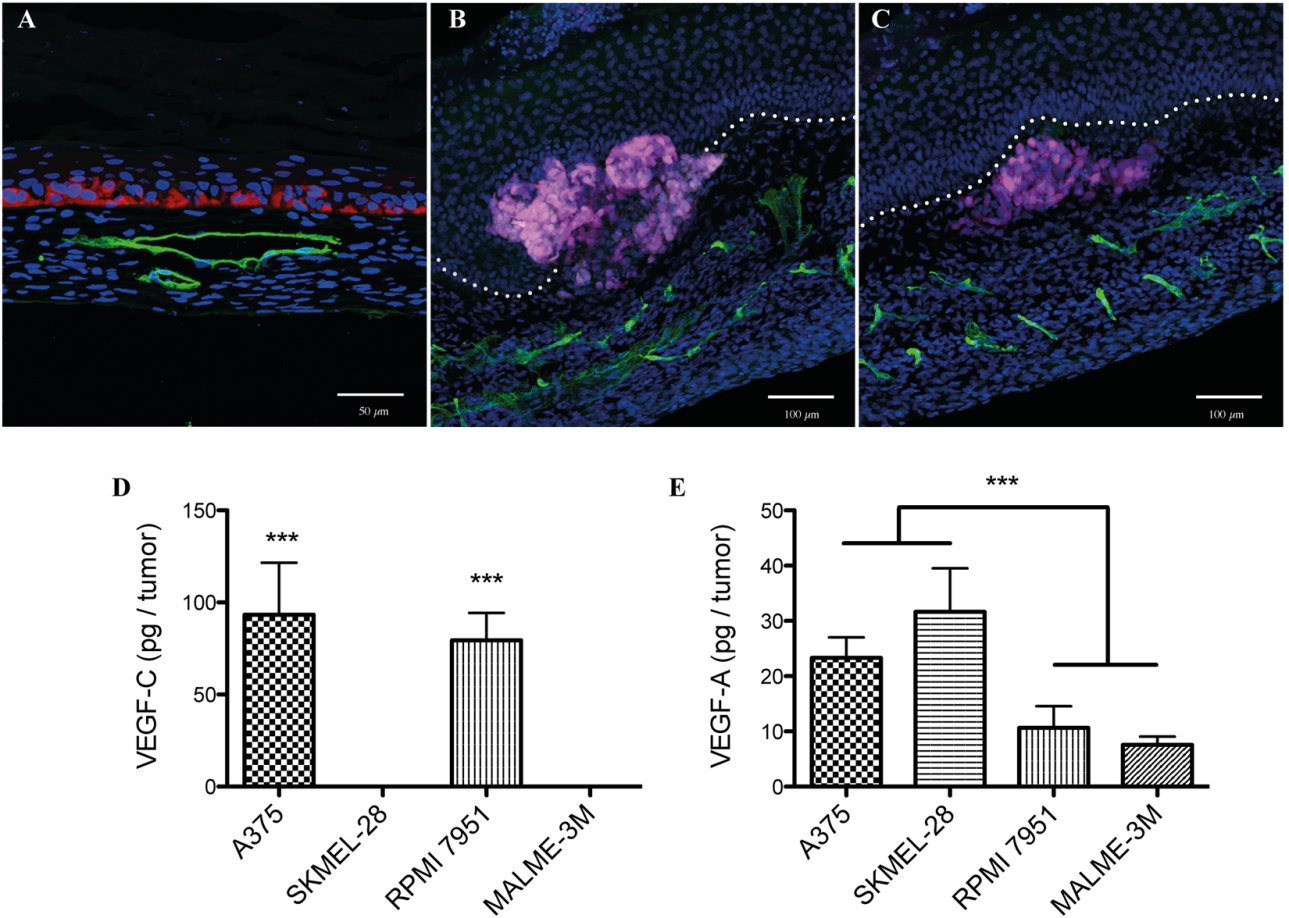
Tumor growth in a tissue-engineered microvascularized environment. (**A**–**C**) Immunofluorescence of Malme-3M melanoma cells (red/pink signal) on (**A**) 8 µm and (**B**,**C**) 70 µm transverse cryosections of the complete melanoma model. Podoplanin staining (green signal) revealed lymphatic capillaries in the dermis. The dermo-epidermal junction is indicated by a white dotted line. Nuclei were counterstained with Hoechst (blue). A375 and RPMI 7951 melanoma cells secreted significant amount of (**D**) pro-lymphangiogenic factor VEGF-C and (**E**) VEGF-A as measured by ELISA in media conditioned by spheroids only. (N = 2 donors, performed in triplicate). ***p-value ≤ 0.001. Statistical analysis: one-way ANOVA with Bonferroni post-test. Scale bars: (**A**) 50 µm, (**B**,**C**) 100 µm.

Melanoma spheroids were rapidly integrated in the epidermis at the dermo-epidermal junction and continued to grow *in vitro* (Suppl. Fig. 3). After 21 days of culture at the air-liquid interface, tumors expanded below the epidermal layer and invaded the dermal compartment, showing combined signs of cellular proliferation, tumor growth and migration (Fig. 4). This spreading was particularly visible for Malme 3 M spheroids, which conserved their ability to secrete melanin and were thus pigmented (Fig. 3H). Depending on the melanoma cell line, the tumors formed masses of different diameters, from 200 µm for the less proliferative (RPMI 7951) to up to a few millimeters for WM983B. All spheroids were easily visible on the reconstructed skin. At the microscopic level, melanoma nodules were visible at the dermo-epidermal junction and were located in close proximity to the lymphatic capillaries (Fig. 4A–C). Crosstalk between the tumor cells and capillary networks is mediated in part by the secretion of different growth factors. Most tumor spheroids produced pro-lymphangiogenic VEGF-C and pro-angiogenic VEGF-A (Fig. 4D,E), as quantified by ELISA. These factors were also detected in media conditioned by the cutaneous tissue (reconstructed skin with or without endothelial cells) (Suppl. Fig. 4). The blood and lymphatic capillaries present in the tumor area seemed to display a slightly more disorganized morphology, which may vary according to the cell lines (Suppl. Fig. 5). A more extensive characterization of the blood and capillary networks morphology (e.g. volume, branching) as a function of the distance from the tumors would be needed to confirm these observations.

**Figure 5:**
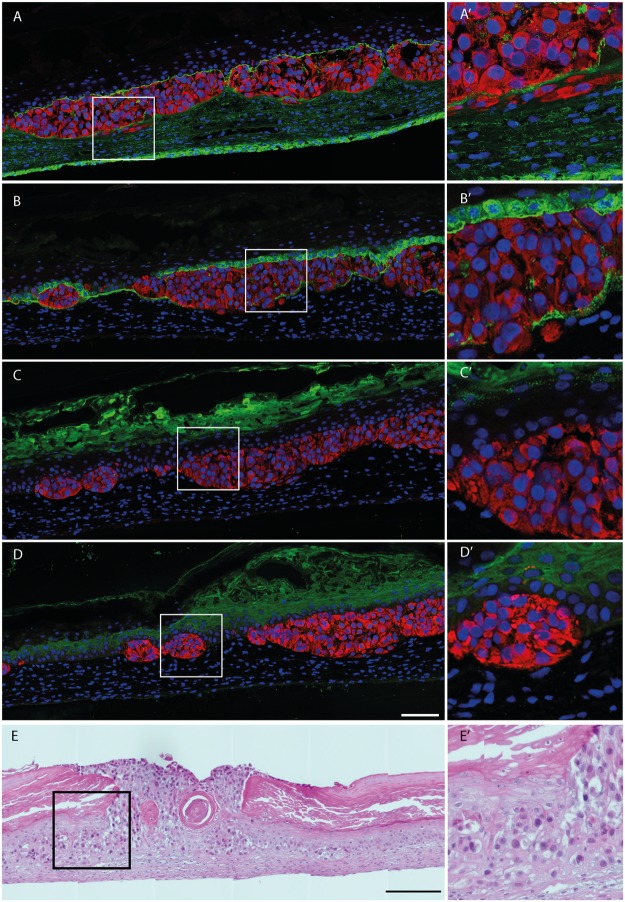
Melanoma spheroids integration into the human reconstructed cutaneous microenvironment. (**A**–**D**) Immunostainings of selected cutaneous proteins (green signal): (**A**) fibronectin staining of the dermis, (**B**) laminin 332 staining indicating the presence of a basement membrane, (**C**) filaggrin and (**D**) involucrin stainings (scale bar: 100 µm). Melanoma cells (SK-MEL 28) are labeled in red following premelanosome protein (PMEL) staining while nuclei are stained with Hoechst (blue signal). (A’–D’) Magnified views of the boxed areas in (**A**–**D**) respectively. (**E**) Hematoxylin and eosin staining of the melanoma model (SK-MEL 28), with (E’) boxed area (Scale bars: 200 µm, boxed areas: 50 µm). Results are representative of two independent experiments, performed in triplicate.

**Figure 6:**
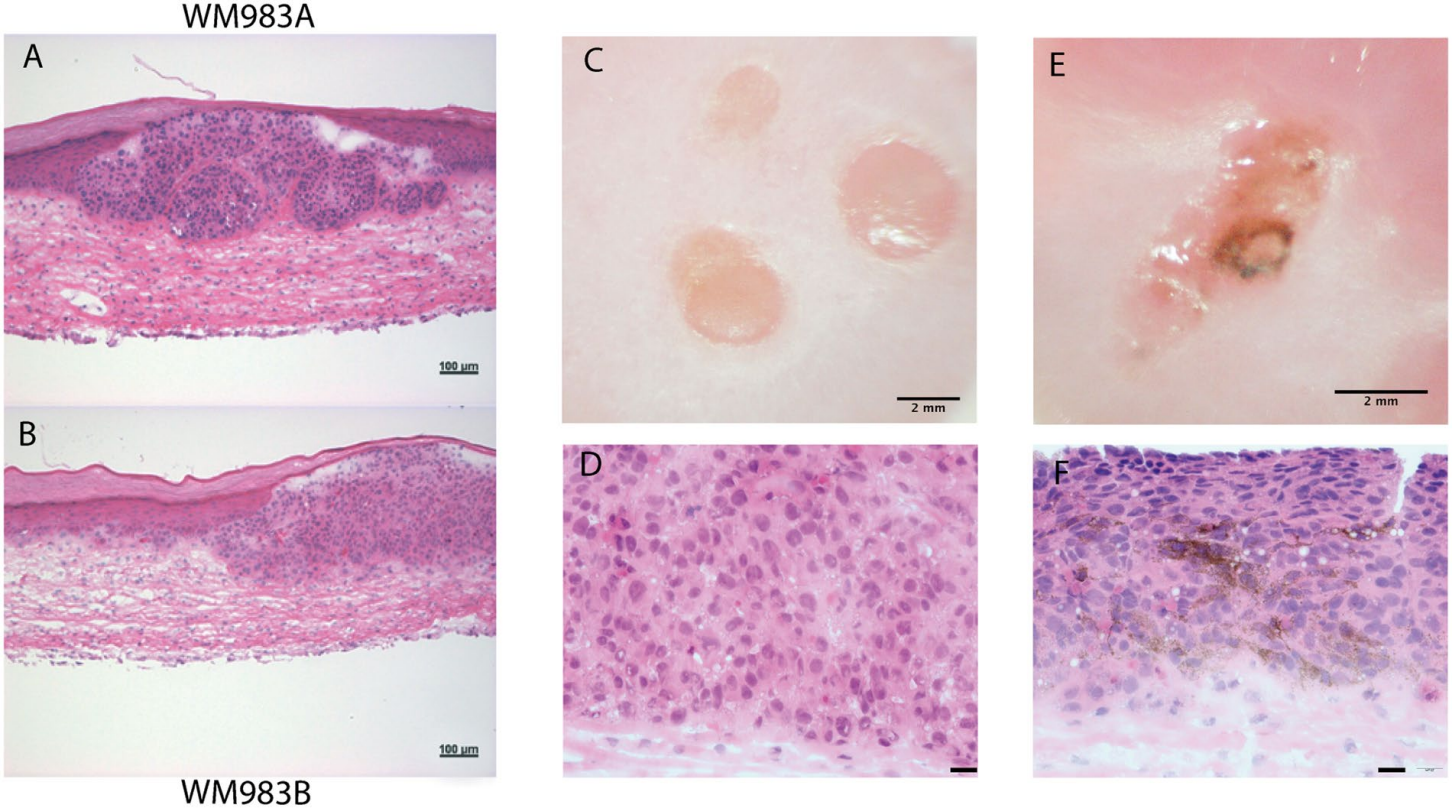
Melanoma tumor morphology is modified by vemurafenib treatment. (**A**) Hematoxylin and eosin staining of WM983A melanoma model revealed a proliferative nodule morphology whereas (**B**) the WM983B model showed a more invasive morphology. (**C**) Macroscopic aspect of non-treated WM983B model with dense tumors and (**D**) corresponding histological appearance after hematoxylin and eosin staining. (**E**) Macroscopic aspect of WM983B model treated with 5 µM vemurafenib for 12 days showed tumor pigmentation, which is also visible on (**F**) hematoxylin and eosin stained tissue sections of the same model. Results are representative of two independent experiments, performed in triplicate. Scale bars: (**A**,**B**) 100 µm, (**C**,**E**) 2 mm, (**D**,**F**) 20 µm.

Clusters of tumor cells were visible in the dermis suggesting the ability of tumor cells to invade this tissue (Fig. 5A–E). A strong labeling of fibronectin (Fig. 5A) was observed in the dermis and around tumors cells. A basement membrane composed of laminin 332 (Fig. 5B), as well as collagen types VII (not shown) and IV (not shown), among others, was detected at the dermo-epidermal junction. It was locally disrupted in close proximity to tumor cells (Fig. 5B). Tumor development seemed to impact the integrity of the epidermis but did not prevent its formation (Fig. 5C–E). Keratinocyte differentiation into a stratified epidermis was confirmed by the detection of filaggrin (Fig. 5C) and involucrin (Fig. 5D), as well as by the histological features (Fig. 5E). WM983A and WM983B, respectively primary and metastatic cell lines from the same patient, presented morphologies representative of their respective origins (Fig. 6A,B). WM983A had a more proliferative morphology, with circular structures (Fig. 6A), whereas WM983B seemed to invade the dermis more efficiently, without a defined border (Fig. 6B).

### Response to a targeted therapy in an organotypic environment

**Figure 7:**
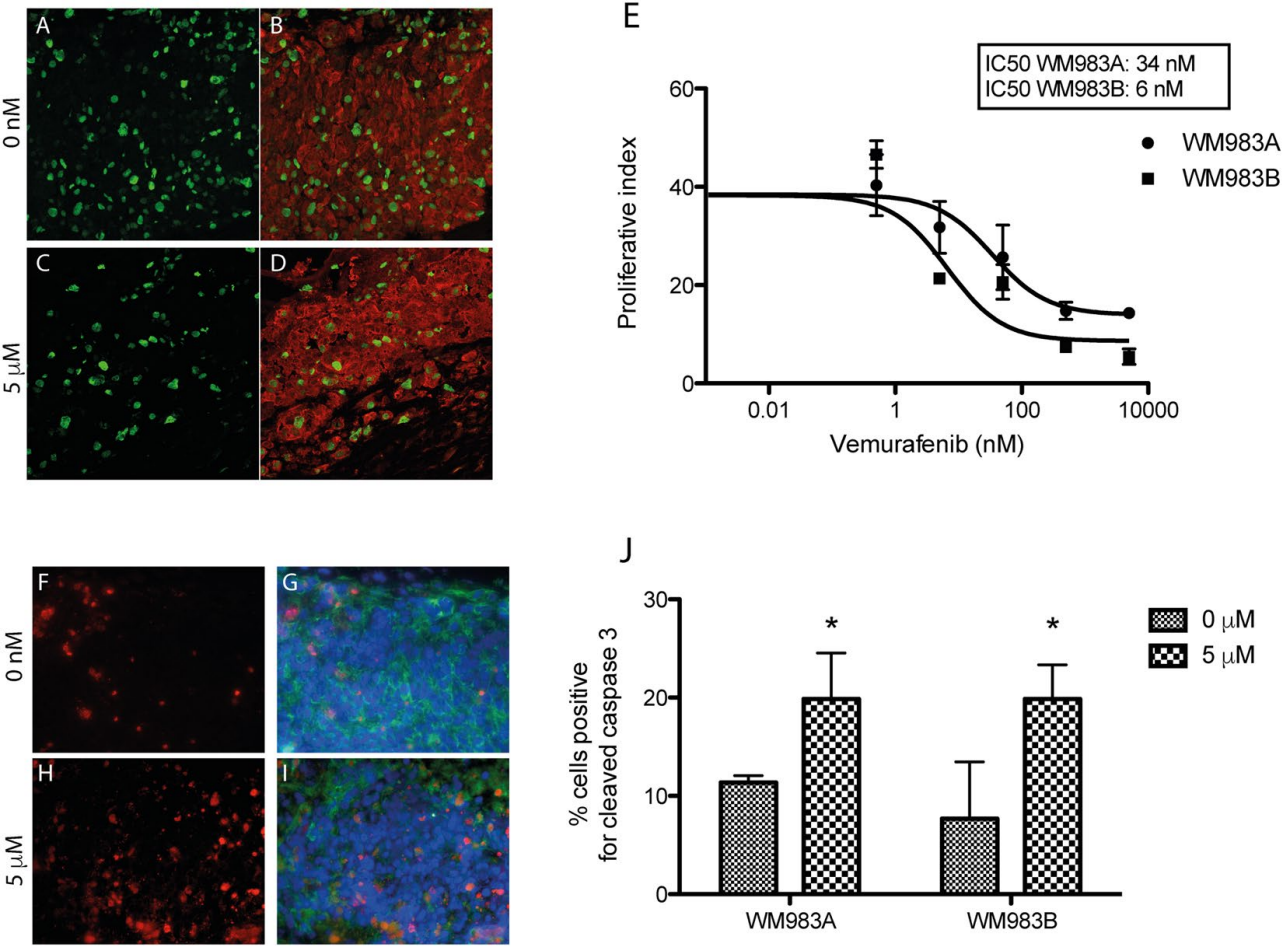
Melanoma cell response to vemurafenib treatment in the 3D microenvironment. (**A**–**D**) Evaluation of melanoma cell proliferation in the model after 12 days of treatment with vemurafenib. Ki67 immunostaining (green signal) is indicative of cell proliferation in the WM983B melanoma tumor (**B**,**D**, red signal, PMEL). (**E**) Proliferative index of WM983A and WM983B cells in the 3D model in response to increasing vemurafenib concentrations. (**F**–**I**) Co-labeling of apoptotic cells after staining for cleaved caspase 3 and tumor marker NG2. (**F**,**H**) Immunostaining for cleaved caspase 3 (red signal) in the melanoma model with (**G**,**I**) immunostaining of melanoma cells (neural/glial antigen 2 (NG-2), green signal and Hoechst, blue signal). (**J**) Quantification of cleaved caspase-3 expressing cells per total number of tumor cells as a function of vemurafenib concentration. (*p ≤ 0.05 as determined by ANOVA followed by a multiple comparison). Results are representative of two independent experiments, performed in triplicate.

The cell lines WM983A and WM983B are both BRAF-V600E positive and are expected to be sensitive to the targeted therapy vemurafenib. The effect of a chronic treatment (12 days, doses ranging from 0 to 5 µM) was assessed on these cell lines using the fully microvascularized melanoma model (Figs 6–7). Untreated WM983B tumors were not pigmented (Fig. 6B,D), but after 12 days of treatment at the highest dose of vemurafenib, dark pigmentation of the tumor periphery was observed, consistent with melanin production (Fig. 6E,F).

Upon treatment, a dose-dependent response was observed at the cellular level, as reflected by the proliferative index (Fig. 7A–E). In vehicle-treated tissues, 40% of tumor cells were proliferative whereas only 5% were proliferating at the higher dose of vemurafenib after 12 days (Fig. 7E). Both cell lines responded to the treatment, but the metastatic WM983B model was slightly more sensitive with a lower IC50 (Fig. 7E). When compared to the melanoma spheroids only, more cells were still proliferative in the complete model (Suppl. Fig. 6), suggesting a potential to study therapeutic resistance. The tumor cell apoptosis was quantified using cleaved caspase 3. After 12 days of treatment at high dose of vemurafenib, apoptosis was significantly increased in the tumors (Fig. 7F–J).

## Discussion

### Creating a melanoma model featuring blood and lymphatic microvascularization *in vitro*

For the first time, we report the production a human reconstructed skin possessing both blood and lymphatic capillaries in a human ECM, and observed the formation of two distinct capillary networks. Using this new reconstructed skin, which do not contain exogenous biomaterial or scaffolding elements, we developed a 3D melanoma model mimicking many attributes of the human *in vivo* microenvironment. The addition of blood and lymphatic capillaries provides an increased possibility of crosstalk between the tumor cells and their environment. This is in agreement with our previous studies showing that either the blood or the lymphatic capillaries obtained within self-assembled tissues are metabolically active and form a network that can easily be modulated by different stimuli. For example, lymphatic capillaries alone in a reconstructed dermis were modulated by VEGF-C and HGF^25^. Compounds like tamoxifen or echistatin were also shown to influence blood capillary formation in a self-assembled skin^39^. Moreover, once grafted, the capillaries quickly connected to the host’s microvasculature^15,39,40^. Here we also show that the capillary networks were metabolically active as indicated by the secretion of ANG-2 and CCL21.

Tumor models reported in the literature are based on the co-culture of various endothelial cells (either HUVEC or HMVEC) with tumor cells. Many recent developments have been achieved to facilitate the *in vitro* study of tumor-associated angiogenesis. For example, ECM density can be controlled in a 3D environment in order to ascertain tumor progression and angiogenic development^31^. However, HUVEC are still often used for microvascularization assays, even though their angiogenic properties differ from native skin vessels^41^. Other models are based on spheroids co-cultured with endothelial cells. They represent high-throughput models but lack the ECM-rich native tissue structure^42–44^. A recent study described a microfluidic system for the co-culture of tumor cells, stromal cells, HUVEC and LEC^45^. This model takes into account the modulation of the capillary flow, but do not mimic the more global tissue organization. This type of model provides useful complementary data when compared to our melanoma model. Angiogenesis and lymphangiogenesis are not only modulated by tumor cells but also by the surrounding stromal and epithelial cells, which is why organotypic models such as ours are currently developed.

### Development of tumors in a relevant human cell-based microenvironment

We observed different responses of the surrounding cell types indicative of cellular crosstalk with the tumor. First, keratinocytes were altered near the spheroid masses, suggesting an impact of the tumor on epidermal integrity by either mechanical forces or paracrine effects. The presence of keratinocytes also allowed the formation of the basement membrane, a key element in the study of melanoma progression. Tumor spheroids added to the model had a diameter superior or equal to 250 µm and were thus submitted to nutrient gradients and core hypoxia^46,47^, mimicking *in vivo* tumor organization^48^. But on their own, they are not exposed to a microenvironment recapitulating cell-cell and cell-matrix interactions, which is provided by their incorporation in engineered skin constructs. Our work demonstrated the inclusion of the melanoma cells between a differentiated epidermis and a dermis produced by the deposition of human ECM components. Fibroblast-derived ECM facilitates physiological interactions between tumor cells and dermal components, mimicking *in vivo* intercommunication^33^. It is now recognized that fibroblasts impact tumor cell proliferation and invasion depending of the donor’s age through their secretion profile^32,49,50^. Different models also incorporate stromal cells to study the effect of their secretome but few are based only on ECM produced and organized by the human fibroblasts, combining both effects. Traditional tumor models are based on rat or bovine collagen^7,32^, fibrin^31^, alginate or hyaluronic acid^51,52^. Keratinocytes are also involved in melanoma progression^53^ and their presence is essential to study cellular responses to compounds in a complete tumor microenvironment. In our melanoma model, VEGF-C was secreted by the cutaneous tissue, likely contributing to the long-term maintenance of the capillary networks (stable at least 3 weeks at the air-liquid interface). Pro-angiogenic and pro-lymphangiogenic molecules are also produced by melanoma spheroids, which can modulate the networks. CCL21 is secreted by LEC and is a chemoattractant involved in immune cell trafficking and tumor cell migration towards lymphatic vessels^54^. It was only detected in presence of EC incorporated into the microvascularized model, indicating LEC secretory activity and possible crosstalk with the tumor^55^. In addition, ANG-2 secretion confirmed the activity of the LEC counterpart, the BEC^56^. Furthermore, to study the role of hypoxia in cell invasion and vessel remodeling, this model could be exposed to a hypoxic environment mimicking the limited oxygenation of tumors *in vivo*.

### Drug response in a 3D human microenvironment

In response to a chronic treatment with vemurafenib, the proliferation of tumor cells decreased strongly (4-fold reduction) but some proliferative tumor cells remained even after 12 days of treatment. These cells were often located near the fibroblasts, suggesting an effect of the microenvironment. It is expected to observe a lower proliferation rate in the inferior part of the tumor and at its periphery, but the opposite effect was noted. Peripheral cells were still proliferative but started to produce pigmentation, showing a clear response to treatment. Indeed, vemurafenib has been shown to induce melanogenesis in melanoma cells, suggesting a transition towards a more differentiated state^57^. When compared to the response of isolated spheroids to treatment, the proliferation rate in the 3D model after treatment was higher, indicating the importance of the TME. This suggests the model could also be a valuable tool to study therapeutic resistance by monitoring tissue responses in culture after stopping the treatment.

While it is now clearly established that monolayer cultures do not adequately recapitulate tumor biology for compound testing, the effect of the neighboring cells and tissue architecture is often understated^6^. Different models are now available to study melanoma using reconstructed skin, but most of them are based on exogenous biomaterials enriched with fibroblasts and keratinocytes^7,11,16,58^. Here we present a model with an extracellular matrix secreted by human fibroblasts *in situ*, thus mimicking the matrix invasion by the tumor. Furthermore, the addition of human blood and lymphatic capillaries recreated a more complete microenvironment to study the effects of various compounds on the tumor-endothelium interaction, but also their off-target effects on the surrounding tissue.

### Application to drug discovery

Since it mimics a human *in vivo*-like environment, this unique *in vitro* 3D model could represent an efficient final step for *in vitro* drug testing and become an alternative to animal testing according to the 3R-principle of Russell and Burch^59^, potentially leading to improved clinical translation. As described, the method of production of the model presented in this work requires 5 weeks to obtain a complete model ready for *in vitro* investigations. This low throughput model, by recapitulating key features of epidermal differentiation and capillary assembly, can be an excellent tool to complete and confirm data obtained from high throughput screening using spheroids alone^46,60^. The production time of the dermal compartment can be further reduced by using reseeding methods^61^. Being produced in standard 12 well- and 6 well culture plates, tissues can easily be adapted for partially automatized production, allowing a scale-up and a more high throughput-like use. We established that our melanoma models were stable at least one month in culture after maturation, allowing to investigate both acute and chronic treatment regimens.

The tissue density and model size also need to be considered when designing a preclinical *in vitro* model. Drug delivery and diffusion need to be extrapolated from *in vivo* observations. In this study, our data suggested that even in a dense tissue, vemurafenib can diffuse and affect the tumor proliferation without evident signs of toxicity in the surrounding tissue. Drug penetration could also be studied in this type of model, like it has been done using spheroids^62^.

Instead of melanoma cell lines, it would be possible to use primary tumor cells from patients to test compounds and select treatments for personalized medicine. Indeed, a minimal amount of tumor cells are required to form spheroids^63,64^, and cutaneous cells can easily be obtained from a small biopsy to produce skin. Spheroids and organoids are already tested as tools to optimize treatment for each patient, but the role of TME is not recapitulated in these models. Reconstructed skin can easily be produced with the patient’ cells^36^ and thus patient-derived organoids could be included to produce an autologous model to evaluate the best treatment option. The self-assembly method has been applied to the engineering of different tissues such as cornea^65^, bladder^66^, adipose tissue^67^, and bone^68^, and accordingly would be readily amenable for an application to other types of neoplasia such as bladder cancer^69^.

## Material and Methods

### Ethical clearance statement

Primary fibroblasts, human microvascular endothelial cells (HMVEC) and keratinocytes were isolated from skin biopsies of either young (20 to 50 days old) or adult donors (18 to 38 years old). All donors or donors’ legal representative(s) provided their informed consent in writing. Protocols were approved by the institutional ethical review board of the CHU de Québec-Université Laval Research Center and performed in accordance with the Helsinki Declaration of 1975, as revised in 2008.

### Cell isolation

Fibroblasts, keratinocytes and HMVEC were obtained from resected skin of healthy donors, either from adult skin for fibroblasts and keratinocytes (breast, abdominal skin from female donors) or newborn foreskin for HMVEC. Fibroblasts and keratinocytes were extracted as previously described^70,71^. Briefly, the epidermis was separated from the dermis using thermolysin (Sigma-Aldrich, Saint-Louis, MO, USA). Fibroblasts were obtained after collagenase digestion. HMVEC were obtained from foreskin dermis by extrusion and expanded on gelatin-coated flasks. CD31-positive cells were selected by immunomagnetic purification^40^. Cultured cells were cryopreserved and thawed prior to amplification for tissue reconstruction. Fibroblasts and HMVEC were used at passage 4 and keratinocytes at passage 2.

Melanoma cell lines A375, Malme 3 M, RPMI 7951 and SK-MEL 28 were obtained from ATCC (Manassas, Virginia, USA). A375 and SK-MEL 28 are derived from a primary site whereas Malme 3 M and RPMI 7951 are derived from a metastatic site. WM983A and WM983B (Wistar Institute, Philadelphia, Pennsylvania, USA) are derived respectively from the primary and metastatic site of the same patient. All these cell lines present the BRAF-V600E mutation. A375 cells were used between passages 168 and 169, Malme 3 M between passages 30 and 32, RPMI 7951 between passages 22 and 24, and SK-MEL 28 between passages 30 and 32. WM983A and WM983B were used at passages 30–32 and 63–65, respectively.

### Cell culture

Culture of fibroblasts was performed in Dulbecco-Vogt modification of Eagle’s medium (DMEM, Invitrogen, Waltham, Massachusetts, USA) supplemented with 10% Fetal Bovine Serum (FBS) (Hyclone, GE Healthcare, Little Chalfont, United Kingdom), 100 U/mL penicillin (Sigma-Aldrich, Saint-Louis, MO, USA) and 25 µg/mL gentamicin (Schering, Kenilworth, New Jersey, USA). Keratinocytes were grown in co-culture with irradiated 3T3 murine fibroblasts in a 3:1 DMEM-Ham’s F12 medium supplemented with 5% newborn calf serum (FetalClone II, HyClone), 10^−10^ M cholera toxin (Sigma-Aldrich, Saint-Louis, Missouri, USA), 5 mg/mL insulin (Sigma-Aldrich), 10 ng/mL human epidermal growth factor (Austral Biologicals, San Ramon, CA), 0.4 mg/mL hydrocortisone (Calbiochem, San Diego, CA, USA) and 100 U/mL penicillin (Sigma-Aldrich) and 25 µg/mL gentamicin (Schering). HMVEC were grown in EGM-2MV medium (Lonza, Basel, Switzerland). A375, RPMI 7951, SK MEL-28 and Malme 3 M were amplified in complete DMEM media like the fibroblasts, whereas WM983A and WM983B cells were cultured in RPMI 1640 supplemented with 10% FBS (Hyclone), 100 U/mL penicillin (Sigma-Aldrich) and 25 µg/mL gentamicin (Schering).

### Flow cytometry

Flow cytometry was performed using a FACSCalibur apparatus (Becton Dickinson, Franklin Lakes, New Jersey, USA) and results were analyzed with the CellQuest Pro software (Becton Dickinson). HMVEC were trypsinized and stained in PBS with 1% BSA with directly coupled antibodies: anti-podoplanin Alexa 647 (Biolegend, San Diego, CA, USA) and anti-CD31 Alexa 488 (Biolegend) (Table 1). Corresponding isotypic antibodies were used as controls. Gating strategy was performed according to FSC and SSC parameters. 10 000 events were acquired.

**Table 1:** Primary Antibodies used for immunolabeling.

Antibody	Host	Type	Company	Catalog number	Dilution
P-MEL	Rabbit	IgG	Thermo Fisher	PA5-32491	1/100
CD31	Sheep	IgG	R&D Systems	AF806	1/100
Podoplanin	Mouse	IgG1	Angio-Bio Co.	11-003	1/50
LYVE-1	Rabbit	IgG	Fitzgerald	70R-LR003	1/100
CD31 Alexa 488	Mouse	IgG1,*κ*	Biolegend	303109	1/200
Podoplanin Alexa 647	Rat	IgG2a,𝜆	Biolegend	337007	1/200
Ki67	Mouse	IgG1,*κ*	BD Pharmingen	556003	1/700
Cleaved Caspase 3	Rabbit	IgG	Millipore	PC679	1/100
Fibronectin	Mouse	IgG1	ATCC hybridoma	CRL-1606	1/100
Laminin 332	Mouse	IgG1	Millipore	MAB19562	1/100
Collagen type VII	Mouse	IgG	Millipore	MAB1345	1/200
Filaggrin	Mouse	IgG1,*κ*	Abcam	Ab3137	1/500
Involucrin	Mouse	IgG1	Sigma	I9018	1/1000

### Tumor spheroid formation

Tumor spheroids were formed using GravityPLUS^TM^ plates (graciously provided by InSphero AG, Schlieren, Switzerland) according to the hanging drop method. Each spheroid was formed of 500 to 4 000 cells depending on each cell line proliferation rate (Suppl. Table 1). After 5 days of aggregation, spheroids were either collected and added to the 3D model or cultured in GravityTRAP^TM^ plates. Conditioned media was collected after 48 hours in the GravityTRAP^TM^ plate, and frozen until the ELISA assays were performed.

### Skin tissue engineering

Skin substitutes were produced using the self-assembly method^15,72^. Fibroblasts were seeded at 12 000 cells/cm^2^ in 12 well-plates (Corning, New York, NY, USA) in presence of anchor papers to prevent contraction and provide easy handling^30,73^. They were cultured for 21 days in complete DMEM with 50 µg/mL sodium L-ascorbate (Sigma-Aldrich) to allow the formation of cell sheets. The culture medium was changed three times a week. Three cell sheets were used to form the dermal compartment of the melanoma model. After 21 days, 10 000 HMVEC/cm^2^ were seeded on top of two individual cell sheets (Fig. 1). The medium was then replaced by a 1:1 mix of EGM-2MV and complete DMEM with 50 µg/mL sodium L-ascorbate (Sigma-Aldrich). In parallel, melanoma spheroids were added to a third individual cell sheet (up to 4 spheroids per sheet). Three days later, keratinocytes were added to the cell sheets featuring the melanoma spheroids (200 000 keratinocytes/cm^2^). The medium was replaced by complete DMEM-Ham’s F12 supplemented with 50 µg/mL sodium L-ascorbate (Sigma-Aldrich) for the cell sheet seeded with keratinocytes and media changes were then performed daily until day 28. At day 28, cell sheets were lifted and superposed: the keratinocytes/melanoma containing cell sheet was placed on top of two cell sheets seeded with microvascular cells. The resulting construct (melanoma model) was then placed at the air-liquid interface to induce keratinocyte differentiation. Medium used during culture at the air-liquid interface consisted of 3:1 DMEM-Ham’s F12 media supplemented with 5% newborn calf serum (FetalCLone II, HyClone), 10^−10^ M cholera toxin (Sigma-Aldrich), 5 mg/mL insulin (Sigma), 0.4 mg/mL hydrocortisone (Calbiochem), 50 µg/mL sodium L-ascorbate (Sigma-Aldrich), 100 U/mL penicillin (Sigma-Aldrich) and 25 µg/mL gentamicin (Schering). The model was produced under 8% CO_2_ at 37 °C.The model was considered mature after 10 days at the air-liquid interface (formation of a fully stratified epidermis and of two distinct capillary networks) and was then ready for further analyses. Tumor development and tissue organization were assessed after 21 days of culture at the air-liquid interface.

### Histology

After 21 days of maturation or after treatment, tissues were harvested and fixed with 3.7% buffered formalin at room temperature. They were then processed and embedded in paraffin. Paraffin-embedded tissue sections (5 µm) were stained with hematoxylin and eosin to reveal cell nuclei (purple), cytoplasm (red/pink) and extracellular matrix in light pink (performed in triplicate for each experiment)^74^. Bright field images were acquired using an Axio Imager M2 microscope (Zeiss, Toronto, ON, Canada) equipped with an Axiocam ICc1 camera (Zeiss) and the Zen software (Zeiss). Tile images were acquired with 5x magnification and zooms were obtained with simple acquisitions at 20x magnification.

### Immunostaining

Fresh tissue samples were embedded in optimum cutting temperature (OCT) compound, taking care to include a melanoma spheroid. Cryosections (8, 25 or 70 µm) were permeabilized in acetone and then incubated with antibodies (listed in Table 1). Images were acquired with a LSM700 confocal microscope using the Zen software (Zeiss).

### Diameter quantification

The diameter of blood and lymphatic capillaries was measured on non-consecutive cryosections (thickness of 25 µm) stained with antibodies against CD31 (R&D Systems) and podoplanin (Angio-bio Co.). Diameters were measured on 3 tissue sections of 3 biological replicates. Measures were performed using ImageJ software^75^ and are expressed as mean ± standard deviation.

### Cytokine quantification

The cytokine levels were measured by ELISA in media conditioned by spheroids or by complete skin models. Secretion of angiopoietin-2 (ANG-2) was measured in conditioned media incubated 48 hours with the complete melanoma model using a DuoSet ELISA kit (R&D Systems, Minneapolis, MN). Quantification in spheroid supernatants (48 h) was performed during their culture in GravityTRAP^TM^ plates (InSphero AG, Schlieren, Switzerland) using VEGF-A and VEGF-C DuoSet ELISA kits (R&D Systems). Supernatants were frozen and kept at −80 °C. CCL21 quantification was performed using a DuoSet ELISA kit (R&D systems) on a 48 h supernatant previously concentrated using 0.5 mL Amicon Ultra 10 K centrifugal filter units (Millipore, Billerica, MA, USA). Medium incubated without cells was used as a negative control.

### Vemurafenib treatment

Treatment started after 10 days of culture at the air-liquid interface. Vemurafenib (PLX4032, MedChem Express, Monmouth Junction, NJ) was resuspended in dimethyl sulfoxide (DMSO). Final concentrations ranged from 0.5 nM to 5 µM. Vemurafenib was added to culture medium at each medium change, 3 times a week, for 12 days. Final DMSO concentration was below 0.1%. Controls were treated with the same DMSO concentration. After 12 days of treatment, tissues were collected, and tumors were embedded in OCT or prepared for histology. Proliferation and apoptosis were quantified on cryosections (non-serial sections, 5 µm). Two independent experiments were performed (n = 4). Melanoma spheroids were treated and processed in parallel with the same protocol in GravityTRAP^TM^ plates.

### Proliferation and apoptosis quantification

In the 3D melanoma model, proliferation was assessed using immunofluorescent staining of Ki67 and Hoechst. Proliferative rates were obtained by counting melanoma cell nuclei positive for Ki67 (BD Pharmingen) compared to the total number of nuclei in the tumor (proliferation index). Tumor cells were identified using an anti-premelanosome protein (PMEL) antibody (Thermo Fisher Scientific). Apoptotic cells were identified using an anti-cleaved caspase 3 antibody (BD Pharmingen). Images were acquired with a LSM700 confocal microscope using the Zen software (Zeiss). Cell counts were performed using the ImageJ software with the cell counter plugin on three regions of 320 × 320 µm^75,76^.

### Statistical analysis

All statistical analyses were performed using GraphPad Prism (version 5.0a). Experiments were performed 2 to 3 times, with 3 biological replicates, and 6 cell lines (except for vemurafenib treatment for which two paired cell lines were used). For each statistical analysis (either t-test or ANOVA with multiple comparison), p-value is indicated as followed: * indicates a p-value ≤ 0.05, ** indicates a p-value ≤ 0.01, *** indicates a p-value ≤ 0.001.

## Electronic supplementary material

Supplementary information
