# Supplementary material for: Tissue-engineered 3D melanoma model with blood and lymphatic capillaries for drug development
Source: Sci Rep. 2018 Sep 4;8:13191. doi: 10.1038/s41598-018-31502-6 (PMC6123405; doi:10.1038/s41598-018-31502-6)
Supplement: Supplementary file 1 — Supplementary information [file 41598_2018_31502_MOESM1_ESM.docx]

Tissue-engineered 3D melanoma model with blood and lymphatic capillaries for drug development

Supplementary information

Authors:

Jennifer Bourland^1^, Julie Fradette^1^, François A. Auger^1,^*

Centre de recherche en organogénèse expérimentale de l’Université Laval / LOEX, Québec, Qc, Canada; Division of Regenerative Medicine, CHU de Québec – Université Laval Research Center, Québec, Qc, Canada; Department of Surgery, Faculty of Medicine, Université Laval, Québec, Qc, Canada


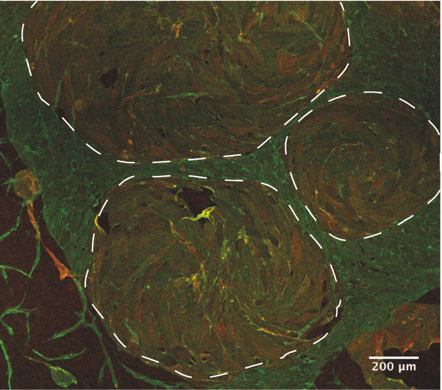


Supplementary Figure 1: Organization of human microvascular endothelial cells (HMVEC) in distinct colonies on a fibroblast cell sheet. HMVEC formed large colonies comprised of LEC positive for podoplanin (red signal) and CD31 (green signal), surrounded by BEC (CD31, green signal). LEC colonies are indicated by dotted lines. Scale bar: 200 µm.


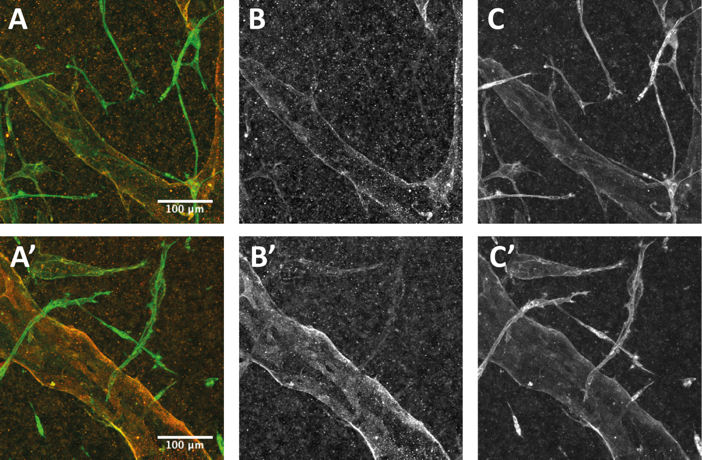


Supplementary Figure 2: LYVE-1 staining of lymphatic structures in the reconstructed skin.

(A-A’) Merge of CD31 (green signal) and LYVE-1 (red signal) immunostaining of two distinct samples. (B-B’) LYVE-1 staining. (C-C’) CD31 staining identifies both blood and lymphatic structures. Scale bars: 100 µm.


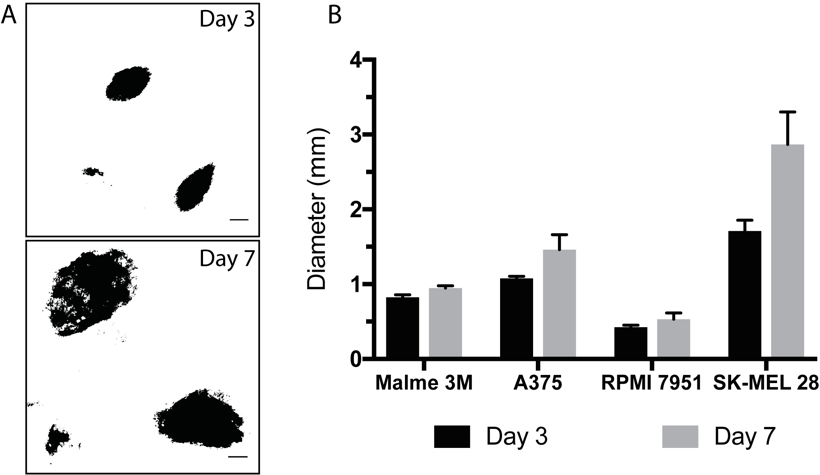


Supplementary Figure 3: Tumor growth in the melanoma model. (A) Evolution of SK-MEL 28 tumors in the 3D melanoma model after 3 and 7 days at the air-liquid interface. This is a representative measure of tumor area after applying a threshold of fluorescence intensity to whole mount immunostaining (Scale bars: 1 mm). (B) The average diameter of the tumors over time was quantified after day 3 and day 7 at the air-liquid interface.


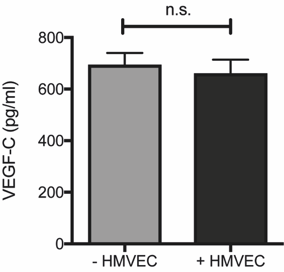


Supplementary Figure 4: Production of VEGF-C by the reconstructed skin. Reconstructed skin composed of fibroblasts and keratinocytes (-HMVEC, grey) secreted a similar amount of VEGF-C compared to the microvascularized skin substitute (+HMVEC, in black). Statistical analysis using a t-test.


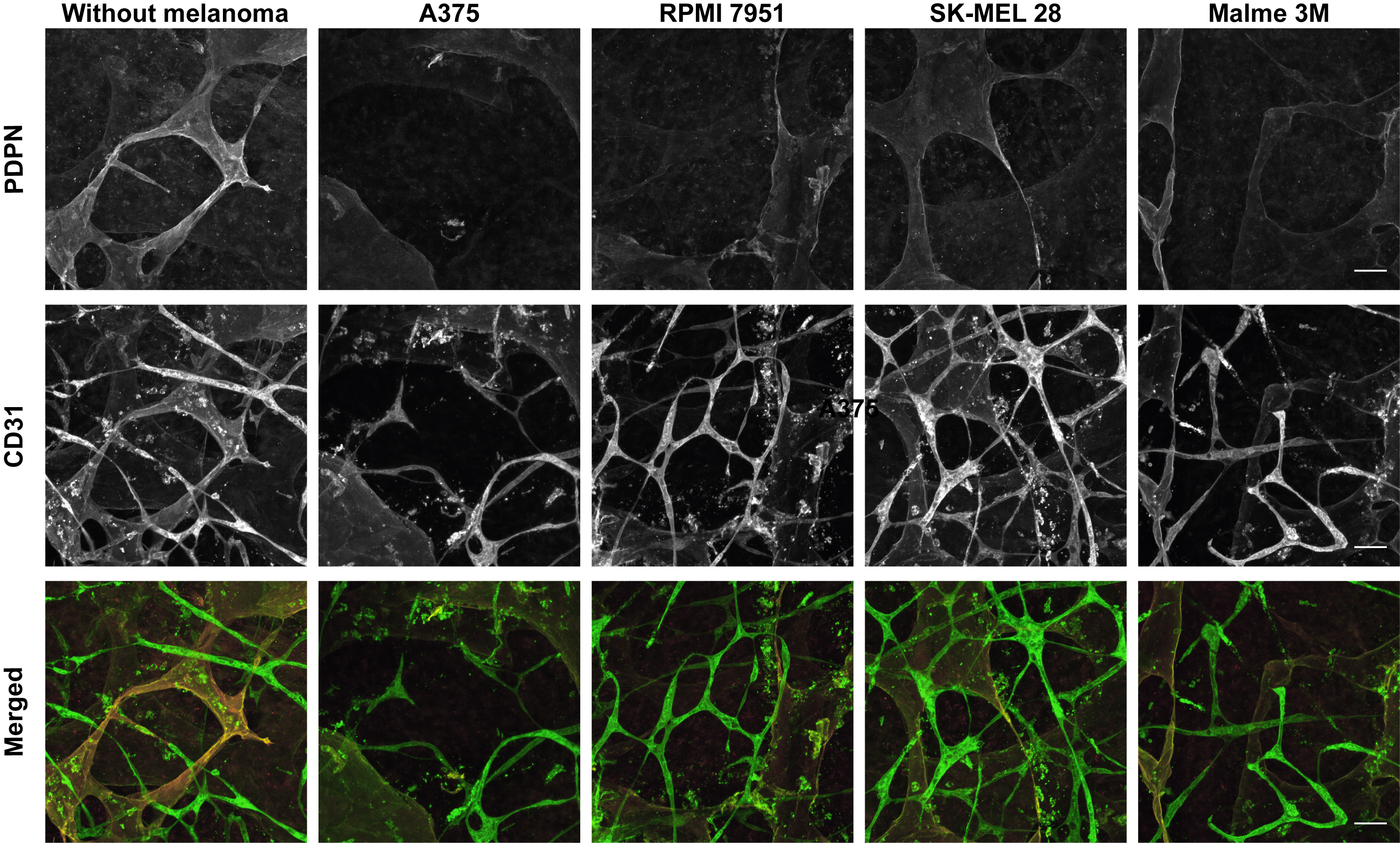


Supplementary Figure 5: Vascular networks in proximity to the tumor area in the 3D model. Blood and lymphatic vessels were observed in absence of tumor cells and below tumors formed by the cell lines A375, RPMI 7951, SK-MEL 28 and Malme 3M as shown by podoplanin (PDPN) staining for lymphatic vessels, and by pan-vessel CD31 staining. Podoplanin is indicated by the red signal in the merged picture while CD31 is indicated in green. The staining was performed on whole mount staining analyzed by confocal microscopy. Scale bars: 40 µm.


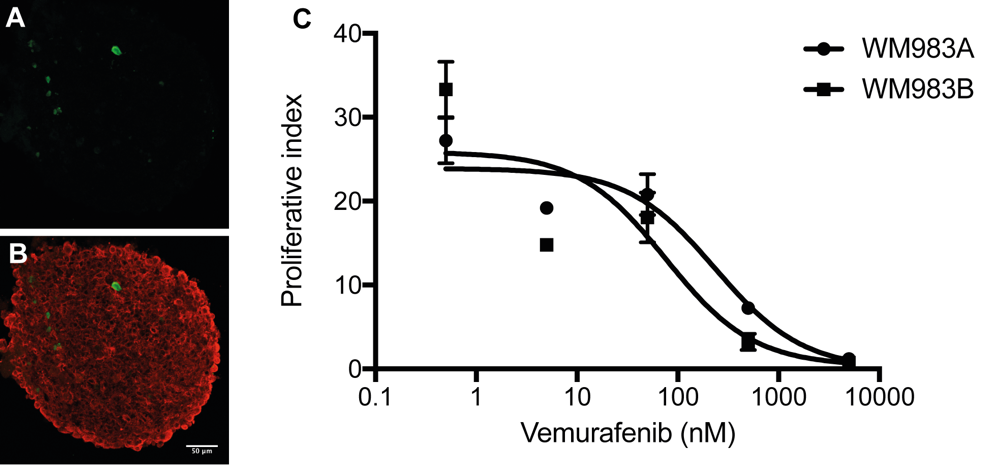


Supplementary Figure 6: Response of melanoma spheroids to chronic vemurafenib treatment. (A) Immunostaining against Ki67 (green signal) of WM983A spheroid treated for 12 days with 5 µM Vemurafenib and (B) merged signal with P-MEL staining (red signal). (C) Quantification of proliferative index in melanoma spheroids after 12 days of treatment in GravityTRAP^TM^ plates.

**Supplementary Table 1: Characteristics of cell lines in the 3D melanoma model**

| *Cell line* | *Origin* | *Initial number of cells for MT formation* | *Tumor Growth*  *Level** | *Features in the 3D model* |
| --- | --- | --- | --- | --- |
| A375 | Malignant melanoma primary site | 500 | Important | Important proliferation leading to the formation of multiple superficial nodules. |
| Malme 3M | Lung metastasis | 1 000 | Medium | Formation of pigmented tumors, important alteration of the epidermis and lateral spreading. |
| RPMI 7951 | Lymph node metastasis | 4 000 | Limited | Very limited growth with the formation of small nodules in the upper part of the dermis. Most invasive cell line. Nodules contain laminin 332. |
| SK-MEL 28 | Malignant melanoma | 500 | Medium | Large macroscopic tumors composed of well-defined nodules surrounded by a basement membrane. |
| WM983A | Primary site | 4 000 | Important | Large tumors composed of proliferative structures. Nodules are well defined. |
| WM983B | Lymph node metastasis (from WM983A) | 4 000 | Important | Large tumors similar to WM983A but with a less defined border, with cells detaching from the nodule and seem more prone to invade the dermis. |

MT: microtissues,

*Growth level: indicates the propension of the melanoma nodules to proliferate in the reconstructed skin.
